# Safe system approach to preventing cyclist fatalities: safety by design for urban and rural environments

**DOI:** 10.1186/s40621-025-00621-w

**Published:** 2025-10-24

**Authors:** Tanya Charyk Stewart, Allison Pellar, Moheem Halari, Kevin McClafferty, Pascal Verville, Michael Pickup, Douglas Fraser, Jason Gilliland, Michael Shkrum

**Affiliations:** 1 Department of Pathology and Laboratory Medicine, Schulich School of Medicine and Dentistry, Western University, London, ON Canada; 2 Motor Vehicle Safety Research Team, Schulich School of Medicine and Dentistry, Western University, London, ON Canada; 3 Department of Pediatrics, Schulich School of Medicine and Dentistry, Western University, London, ON Canada; 4 London Health Sciences Centre, London, ON Canada; 5 Transport Canada, Toronto, ON Canada; 6 Ontario Forensic Pathology Service, Forensic Services and Coroner’s Complex, Toronto, ON Canada; 7 Department of Clinical Neurological Sciences, Schulich School of Medicine and Dentistry, Western University, London, ON Canada; 8 Department of Geography, Western University, London, ON Canada; 9 Research Park, University of Western Ontario, 999 Collip Circle, Room LL02, London, ON N6G 0J3 Canada

**Keywords:** Cyclists, Fatalities, Motor vehicle collisions, Injury prevention, Urban environment, Rural environment, Road safety

## Abstract

**Background:**

Cyclists are vulnerable road users, with preventable deaths increasing by 48% over the past decade. This study aimed to review the epidemiology of cyclist fatalities to identify risk factors for targeted interventions through a safe system approach, with a focus on urban and rural environments.

**Methods:**

Data on fatal cyclist and motor vehicle collisions (CMVC) and injuries were collected from the Office of the Chief Coroner (2013-19), including selected crash investigations and expert reviews by a multidisciplinary team. Descriptive analyses were conducted, and urban vs. rural CMVC were compared using Pearson chi-square and Mann-Whitney U tests.

**Results:**

There were 83 fatal cyclist collisions (81% male), with 6% children, 13% youth, 69% adults, and 12% seniors (median age = 48, ISS = 75). The head was the most severely injured body region across all age groups (median AIS = 5), except for children, whose thoracic injuries were more severe. Overall, 62% of cyclists were not wearing helmets, and 24% were impaired. Expert review found that 60% of child cyclist fatalities were run over, all of whom were ≤ 6 years. Distractions from cell phones (1%) or headphones (8%) may have contributed to CMVC. Urban collisions (49 cyclists; 59%) accounted for all child deaths and had significantly more collisions involving intersections (57% vs. 6%; *p* < 0.001), low-speed crashes (33% vs. 0%; *p* < 0.001), bike lanes (29% vs. 0%; *p* < 0.001), and heavy vehicles (31% vs. 6%; *p* = 0.006). Rural collisions were associated with higher speeds (> 50 km/h, 94% vs. 49%; *p* < 0.001), dark lighting (44% vs. 4%; *p* < 0.001), and riding on the roadway with traffic (56% vs. 16%; *p* < 0.001). No rural CMVCs had sidewalks or bike lanes (0% vs. 84%; 0% vs. 33%; *p* < 0.001).

**Conclusion:**

Cyclists face severe injury and death risks in both urban and rural settings. A safe system approach recognizes human vulnerability and the inevitability of mistakes. Engineering countermeasures, such as road separation, better lighting in rural areas, traffic calming, and vehicle safety features (i.e., guard rails, advanced headlights, and cyclist detection), support CMVC prevention. Public health campaigns and legislative action, along with equitable implementation across urban and rural areas, facilitate improving cyclists’ safety.

**Supplementary Information:**

The online version contains supplementary material available at 10.1186/s40621-025-00621-w.

## Background

Cycling is an active and sustainable mode of transport and recreational activity that has many health, societal, economic, and environmental benefits including physical activity, inexpensive transportation, as well as reduced greenhouse gas emissions [1, 2]. It is an important means of transportation, recreation and fitness, which became even more popular during the COVID-19 pandemic, given proximity issues with public transportation and a halt put to many other types of recreational activities [3, 4]. Despite all its benefits, cycling can have dire consequences, particularly when cycling occurs on roadways in close proximity to traffic [5]. According to the World Health Organization, 41,000 cyclists die annually in road traffic-related incidents worldwide [1]. 

Unlike motor vehicle occupant injuries, which have been consistently on the decline in North American for several decades, except for increases during the COVID-19 pandemic [3, 6], cycling crashes are on the rise, comprising an increasing proportion of road traffic injuries and deaths [7, 8]. Without the protection of a vehicle, cyclists are a particularly vulnerable road user (VRU), killed at higher rates than other types of road users [9]. The risk of injury and death has been estimated to be higher for those riding a bicycle than for those driving a car on roadways [10]. One study found cyclists to be 12 times more likely to be killed while traveling on roadways compared with motor vehicle occupants [11]. This results in a disproportional overrepresentation of cyclists in serious and fatal road traffic injury when riding on the roadways compared to vehicle occupants [2].

According to National Highway Traffic Safety Administration, the number of preventable cyclist deaths have increased 47.5% over the past decade, with *n* = 749 fatalities in 2013 and a total of *n* = 1,105 cyclist fatalities in 2022 [12]. In Canada, the number of cyclist deaths have remained fairly constant in recent years, with only 2 less deaths in 2022 compared to 2018 (*n* = 46 and *n* = 48, respectively), with the number of cycling fatalities peaking in 2020 (*n* = 51), coinciding with the onset of the COVID-19 pandemic over this 5-year time period [8].

Cycling crashes and injuries do not impact individuals and communities equally. Demographic and socioeconomic factors, along with neighbourhood environments and urban planning features have been found to correlate to cyclist injury [3, 13]. Who you are, where you live and where you cycle impacts your likelihood of crashing and sustaining an injury on a bicycle. An individual’s neighbourhood is increasingly acknowledged as a key factor in determining health, as residential areas have both physical and social characteristics that have the potential to influence a person’s health and well-being [13, 14]. Since place of residence is heavily influenced by social status and ethnicity, the characteristics and features of neighbourhoods may play a significant role in contributing to health disparities [14].

In terms of cycling, low-income and other marginalized groups are less likely to own a vehicle and therefore, rely more on public and active forms of transportation. Previous research using census tracts in Texas found that poverty rates were associated with more frequent trips by bicycle, which in turn lead to an increase in cycling crashes [15]. Inadequate urban planning and access to transportation and cycling infrastructure, (i.e., bike lanes, cycle tracks and shared paths), which differs in urban and rural environments, can significantly disadvantage groups and neighboourhoods, leading to more cycling collisions, injuries and fatalities in those areas [13]. A recent pediatric study found low-income neighbourhoods and areas near major roadways had the highest risk for cycling collision. Additionally, helmet access and use were very low among these children, contributing to high rates of traumatic brain injuries [16]. A safe and equitable transportation system needs to be designed that mitigates crash risk in all communities and protects all including cyclists, a very VRU, via a safe system approach [17, 18]. The key principles of a safe system approach acknowledge that all road users will make mistakes, so the transportation system must be designed to account for these inevitable human errors, with an emphasis on a shared responsibility to prevent serious injury and deaths [17, 18]. 

Cyclists are particularly vulnerable because they lack a vehicle to protect their bodies, which has a finite capacity to withstand the forces experienced in a collision. So, designing safer roads, whether in urban or rural settings, is crucial in minimizing serious injury and deaths. Well-designed roadways can proactively manage vehicle speeds, simplify traffic flow and guide all road users to make safer decisions and reduce the crash forces on individuals to prevent serious injury [18]. 

The objective of this study was to examine the epidemiology of cycling fatalities in Ontario, with a focus on urban and rural environments, to identify key risk factors for targeting through a safe system approach. Potential countermeasures for reducing CMVC will be discussed and recommended, based on best practice in each setting via this holistic, proactive approach [18, 19], which can be used as part of a road safety strategy to mitigate potential crash hazards for cyclists and prioritize safe mobility for these VRU.

## Methods

The study was approved by the Western University Health Science Research Ethics Board (Project ID: 113440; Lawson Health Research Institute approval number: R-19-066). The Board agreed to waive the requirement to obtain informed consent because in accordance with Tri-Council Policy Statement 2, Article 3.7 A as the research involved no more than minimal risk to the participants who were deceased; the alteration to consent requirements was unlikely to adversely affect their welfare; and it would have been impossible to carry out the research and to address the research question properly, given the research design, if prior consent of the participants was required.

### Data

Fatal cyclist collision and injury data were collected from the Office of the Chief Coroner for Ontario from 2013 to 19. Data investigation reports from the Coroner’s Office were reviewed, data abstracted and entered into a VRU injury crash database [20]. These reports were comprised of the coroner’s investigation statement, postmortem examination and forensic toxicology reports, along with reports from police and other agencies. Based on these data, the injuries were summarized and coded with the Abbreviated Injury Scale (AIS), 2015 version and Injury Severity Score (ISS) calculated to determine the overall injury severity [21]. An anonymized version of the crash and injury cyclist data is presented in Additional file 1.

The collision data included vehicle details and type, speed limits, impact speed, intersection type and controls, location type, road surface conditions, lighting, collision configurations, pre-crash and avoidance action, with VRU kinematics, position, action, contact and runover status, as well as other details on the VRU and driver of the vehicle [20]. A select subset of all VRU cases (43/703 = 6.1%) underwent in-depth prospective and retrospective field investigations conducted by our crash investigation team. These investigations included collection and review of extensive on-scene collision information and crash reconstruction from local and regional supporting police services. Media information from news articles and collision images were included in the database, used to supplement any data element missing in the Coroner’s database such as vehicle information, confirming weather or lighting conditions, along with scene maps and street view images from Google Maps [22] based on crash location data, as available. These data were important for classifying CMVC as urban or rural, as defined below, as well as determining the presence of infrastructure at the crash scene including bike lanes or the type of crosswalk. Electronic reports were developed for each VRU collision and linked to the injury crash database [20]. Cyclists were the only type of VRU included in this analysis.

Urban and rural crash locations were classified by the following definitions, according to Statistics Canada definitions based on population size and census data. Urban areas were areas situated within a city or town characterized by higher population density and infrastructure development, specifically defined as continuously built-up areas having a minimum population concentration of 1,000 persons and a population density of at least 400 persons per square kilometer based on the previous census [23]. Rural areas have concentrations or densities below the thresholds used to define urban areas [23]. They are areas situated outside urban centers and characterized by lower population density, often with vast expanses of open land areas.

### Analysis

Descriptive analyses were undertaken including counts, proportions, mean, median, standard deviation and interquartile range (25th − 75th quartiles). A subgroup analysis by the classification of the crash location as either urban or rural, as defined above, for cyclist and motor vehicle collisions (CMVC) was undertaken, with proportions and medians compared with Pearson chi square and non-parametric Mann Whitney U tests, respectively. All analyses were performed using IBM^®^ SPSS^®^ Statistics for Windows, Version 29.0.2.0 (Armonk, NY: IBM Corp).

## Results

**Fig. 1:**
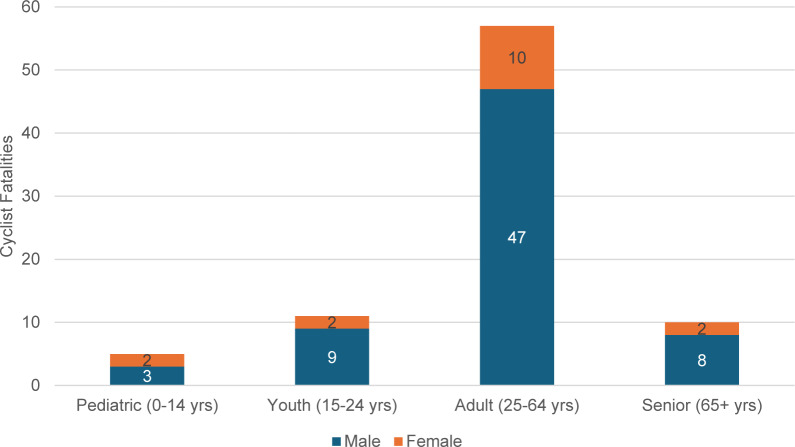
The breakdown of age groups for cyclist fatalities in Ontario

Of the 703 VRU deaths in the VRU crash database, there were 83 unintentional cyclist fatalities over the study period, predominantly male cyclists (*n* = 67, 80.7%). The median (IQR) age was 48 (27.0–58.0) years, with the majority (*n* = 57, 68.7%) of cyclists in the adult age group (25–64 years old). A breakdown of cyclist fatalities by age and sex is presented in Fig. 1. Table 1 provides a summary of crash variables for all cycling fatalities, as well as comparing cycling crashes in a rural versus urban environment.

**Table 1:** Summary of crash variables, for all cycling fatalities, and comparing cycling crashes in a rural versus urban environment

Variables	Total Population Proportion of valued (%)	Rural Proportion of valued (%)	Urban Proportion of valued (%)	*P* value^1^
TEMPORAL VARIABLES				
Time of Day				0.065
Daytime (6:00–17:59)	49/83 (59.0%)	16/34 (47.1%)	33/49 (67.3%)	
Day of Week				**0.025**
Weekday (Monday-Friday)	64/83 (77.1%)	22/34 (64.7%)	42/49 (85.7%)	
Weekend (Saturday-Sunday)	19/83 (22.9%)	12/34 (35.3%)	7/49 (14.3%)	
Seasons				0.175
Spring (MAM)	11/83 (13.33%)	5/34 (14.7%)	6/49 (12.2%)	
Summer (JJA)	42/83 (50.6%)	21/34 (61.8%)	21/49 (42.9%)	
Autumn (SON)	22/83 (26.5%)	7/34 (20.6%)	15/49 (30.6%)	
Winter (DJF)	8/83 (9.6%)	1/34 (2.9%)	7/49 (14.3%)	
CYCLIST STRUCK				
By Vehicle Type				
Car	31/82 (37.8%)	13/32 (40.6%)	18/49 (36.7%)	0.889
Light Trucks & Vans/SUV ^2^	34/82 (41.5%)	18/32 (56.3%)	16/49 (32.7%)	0.065
Heavy Truck/Bus	17/82 (20.7%)	2/32 (6.3%)	15/49 (30.6%)	**0.006**
In Intersection	30/83 (36.1%)	2/34 (5.9%)	28/49 (57.1%)	**< 0.001**
In Crosswalk	11/80 (13.8%)	0/34 (0.0%)	11/46 (23.9%)	**0.002**
Runover/Drag	27/83 (32.5%)	3/34 (8.8%)	24/49 (49.0%)	**< 0.001**
VEHICLE INITIAL ACTION				
Vehicle Going Ahead	65/83 (78.3%)	33/34 (97.1%)	32/49 (65.3%)	**< 0.001**
Slowed or stopped	17/83 (20.5%)	1/34 (2.9%)	16/49 (32.7%)	**< 0.001**
Changing Lanes	1/83 (1.2%)	0/34 (0.0%)	1/49 (2.0%)	0.402
VEHICLE PRE-CRASH MANEUVER				
Vehicle Going Ahead	54/83 (65.1%)	26/34 (76.5%)	28/49 (57.1%)	0.069
Vehicle Turning	14/83 (16.9%)	1/34 (2.9%)	13/49 (26.5%)	**0.005**
Overtaking/Changing Lanes	7/83 (8.4%)	4/34 (11.8%)	3/49 (6.1%)	0.363
Vehicle Lost Control	5/83 (6.0%)	3/34 (8.8%)	2/49 (4.1%)	0.372
Other (Prior Collision or Reversing)	3/83 (3.6%)	0/34 (0.0%)	3/49 (6.1%)	0.142
CYCLING PRE-CRASH ACTIONS				
Riding on Roadway with Traffic	27/83 (32.5%)	19/34 (55.9%)	8/49 (16.3%)	**< 0.001**
While Crossing roadway	15/83 (18.1%)	0/34 (0.0%)	15/49 (30.6%)	**< 0.001**
Riding on Shoulder of Road	11/83 (13.30%)	8/34 (23.5%)	3/49 (6.1%)	**0.044**
Lost Control/Fell	5/83 (6.0%)	1/34 (2.0%)	4/49 (8.2%)	0.402
Riding on Roadway against Traffic	3/83 (3.6%)	3/34 (8.8%)	0/49 (0.0%)	0.065
Other Cyclist Action^3^	20/83 (24.1%)	3/34 (8.8%)	17/49 (34.7%)	**0.007**
CYCLING INFRASTRUCTURE				
Bike Lane or Shared Path Available	16/83 (19.3%)	0/34 (0.0%)	16/49 (32.7%)	**< 0.001**
Struck on Bike Lane/Shared Path	14/83 (16.9%)	0/34 (0.0%)	14/49 (28.6%)	**< 0.001**
Sidewalk Available	41/83 (49.4%)	0/34 (0.0%)	41/49 (83.7%)	**< 0.001**
Struck on Sidewalk	11/83 (13.3%)	0/34 (0.0%)	11/49 (22.4%)	**0.002**
IMPACT SPEED				
Very Low Speed (< = 15 km/h)	16/83 (19.3%)	0/34 (0.0%)	16/49 (32.7%)	**< 0.001**
High Speed (> 50 km/h)	56/83 (67.5%)	32/34 (94.1%)	24/49 (49.0%)	**< 0.001**
Very High Speed (> 70 km/h)	33/83 (39.8%)	21/34 (61.8%)	12/49 (24.5%)	**< 0.001**
Median (IQR) estimated impact speed (km/h)	60 (45–80)	80 (60–80)	50 (15-72.5)	**< 0.001**
ENVIRONMENTAL CONDITIONS				
Road Surface				0.236
Dry	70/83 (84.3%)	26/34 (76.5%)	44/49 (89.8%)	
Wet/Snow	9/83 (10.8%)	5/34 (14.7%)	4/49 (8.2%)	
Unknown	4/83 (4.8%)	3/34 (8.8%)	1/49 (2.0%)	
Weather Conditions				0.200
Clear/Probable Clear	74/80 (92.5%)	27/31 (87.1%)	47/49 (95.9%)	
Rain/Snow/Fog	6/80 (7.5%)	4/31 (12.9%)	2/49 (4.1%)	
Lighting				**< 0.001**
Daylight	50/83 (60.2%)	17/34 (50.0%)	33/49 (67.3%)	
Dark	31/83 (37.3%)	15/34 (44.1%)	2/49 (4.1%)	
Dark with Artificial Light	17/83 (20.5%)	0/34 (0.0%)	11/49 (22.4%)	
Dark Unknown Lighting	3/83 (3.6%)	0/34 (0.0%)	3/49 (6.1%)	
Dusk	2/83 (2.4%)	2/34 (5.9%)	0/49 (0.0%)	
CYCLING BEHAVIUR CONTRIBUTING FACTORS				
Helmet Used	17/73 (38.4%)	12/32 (37.5%)	16/41 (39.0%)	0.894
Lack of Conspicuity/Dark Cyclist^4^	22/34 (64.7%)	13/17 (76.5%)	9/17 (52.9%)	0.151
Impaired/Positive Toxicology^5^	19/77 (24.1%)	11/33 (33.3%)	8/44 (18.2%)	0.127
Type of impairment				0.141
Alcohol only	5/19 (26.3%)	1/11 (9.1%)	4/8 (50.0%)	
THC only	8/19 (42.1%)	5/11 (45.5%)	3/8 (37.5%)	
Multiple Drugs	6/19 (31.6%)	5/11 (45.5%)	1/8 (12.5%)	
BAC^6^ Median (IQR) mg/100 mL	147.0 (118.0-268.0)	121.00 (118.0-.)	207.50 (79.5-341.5)	1.000

^1^ P value of the difference in proportions between rural and urban crash environments based on Pearson Chi-Square or Fisher’s Exact Test results. Statistically significant results are **bolded**

^2^ Light Trucks & Vans (LTV) = Pick up/Van/Mini Van/SUV (Sports Utility Vehicle)

^3^ Other cyclists’ actions include going ahead, turning, riding on sidewalk, swerving, changing lanes, stopped, pulling away from curb/shoulder, unknown action

^4^ When Conspicuity noted on report

^5^ Reported only when toxicology report was available

^6^ BAC = Blood alcohol concentration

For all cyclists, just over half of cyclist (*n* = 42/83, 50.6%) fatalities occurred in the summer months from June to August, with fall, from September to November, the next most common time of year for cyclist fatalities (*n* = 22/83, 26.5%). Thursday was the most common day of the week for cyclist fatalities (*n* = 15/83, 18.1%), more than double the cyclist fatalities on Mondays (*n* = 7/83, 8.4%), the least common day of the week for these collisions. Fatal cyclist crashes occurred at every hour throughout the day, with 6 o’clock am and pm being the two most common hours of occurrence (*n* = 10/83, 12.0% and *n* = 7/83, 8.4% for 06:00–06:59 and 18:00–18:59, respectively). The majority of fatalities occurred in the daylight (*n* = 50/83, 60.2%), with clear/probable clear weather conditions (*n* = 74/83, 60.2%) and dry (*n* = 68, 81.9%) or probable dry (*n* = 2, 2.4%) road conditions.

Over two-thirds of collisions were at high speeds [> 50 km per hour (km/h); *n* = 56/83, 67.5%], with 39.8% (*n* = 33/83) at very high speeds in excess of 70 km/h. The vehicle was most often a light truck or van (LTV)/SUV (*n* = 34/82, 41.5%), going ahead (*n* = 54/83, 65.1%) or turning (*n* = 14/83, 16.9%). Over one-third of CMVC were intersection-related (*n* = 30/83, 36.1%). The leading cyclist pre-crash actions were riding on the roadway with traffic (*n* = 27/83, 32.5%), crossing the roadway (*n* = 15/83, 18.1%) and riding of the shoulder of the roadway (*n* = 11/83, 13.3%).

**Fig. 2:**
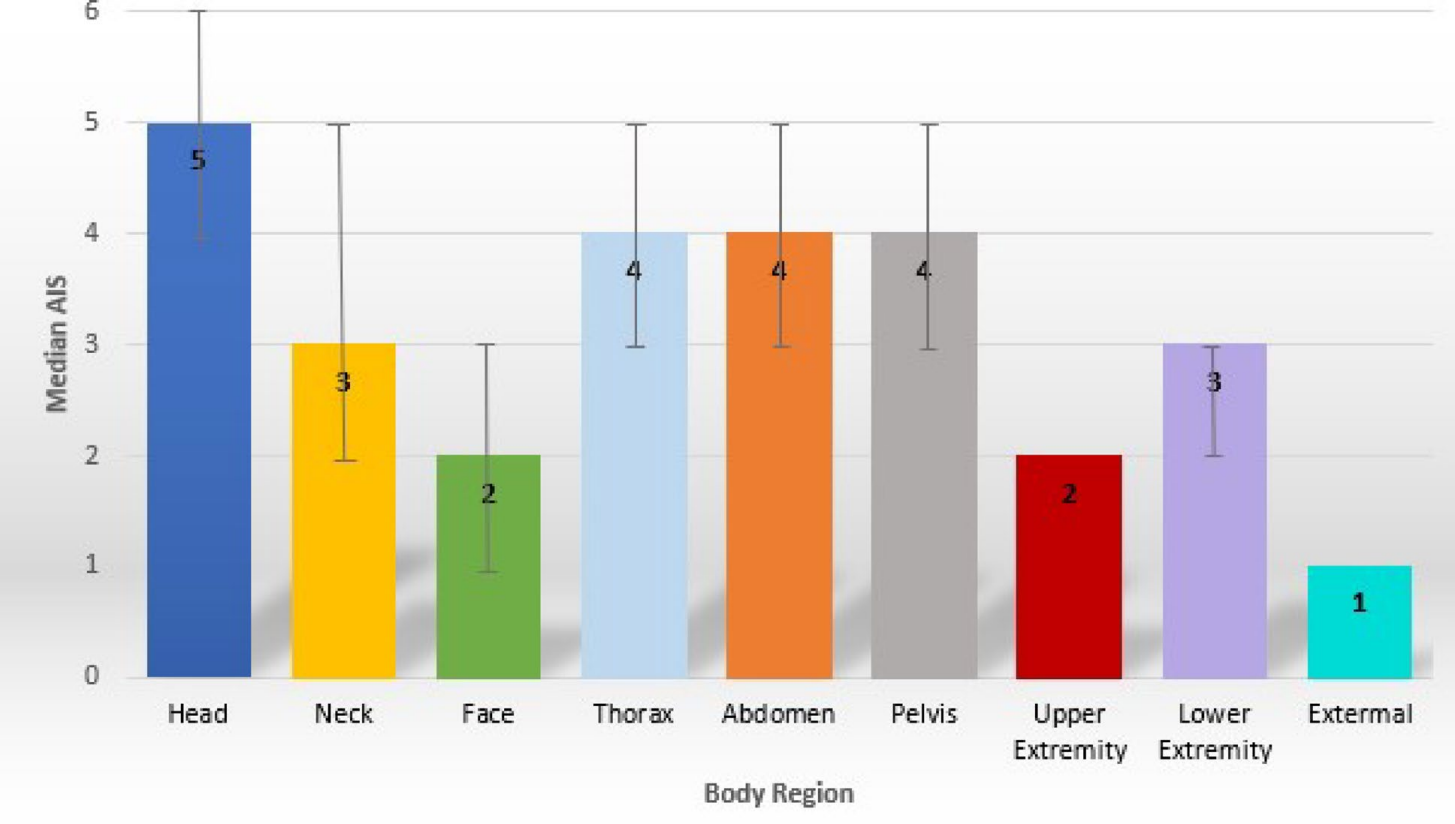
Median Abbreviated Injury Scale (AIS) by body region for all cyclist fatalities in Ontario. An AIS of 1 = minor, 2 = moderate, 3 = serious, 4 = severe, 5 = critical and 6 = maximal, currently untreatable injury [21]. The error bars represent the 1st to the 3rd quartiles of the interquartile range

The median AIS by body region for all cyclist fatalities is presented in Fig. 2. When examining the injuries sustained by cyclists, the head was found to be the most often and most severely injured body region overall with 91.6% of cyclists sustaining a head injury with a median (IQR) AIS of 5 (4–6) representing a critical injury [21]. It was the most severely injured body region for each age group, except for children with thorax (median [IQR] AIS of 4.5 [3.75-5.0]) most severely injured. Expert review found that 60% of child cyclist fatalities were run over, all of whom were ≤ 6 years. The median AIS of the thorax was higher at 5, signifying a critical injury [21], for the children runover compared to those not runover with a median AIS thorax = 3.5 representing a serious to severe injury [21]. The overall injury severity, as measured by the ISS, was very high, with the median (IQR) ISS = 75 (45–75), representing maximum injury [21]. 

A review of possible contributing factors in these deaths found for cyclists with helmet use known, nearly 2/3 of cyclists (62.2%) were not wearing a helmet. Helmet use ranged from 10.0% in youth, 38.8% in adults, 40.0% in children, followed by seniors with the highest helmet use at 60.0%. For those with conspicuity data entered, 64.7% (*n* = 22/34) had a lack of conspicuity including dark clothing and no lights or reflective material. When toxicology reports were available, 24.1% were found to be impaired at the time of the crash, testing positive to a variety of drugs. Of these impaired cases, THC (*n* = 8/19, 41.2%) was the most common drug used, most often taken on its own without other substances. The median blood alcohol concentration (BAC) was 147 mg/100 mL for cyclists positive for alcohol. Distraction from cell phones (*n* = 1/83, 1.2%) or headphones (*n* = 7/83, 8.4%) found at the scene, on or with the cyclist, may have contributed to CMVC.

### Urban versus rural cyclist fatalities

There were 49 (59%) cyclists killed in an urban environment (Table 1). The median (IQR) age was 44.0 (24.0-56.5) years. All child cyclist deaths occurred in an urban environment. Comparing urban CMVC to rural, urban collisions had a significantly higher proportion of collisions involving an intersection and a crosswalk (57.1% vs. 5.9% and 23.9% vs. 0.0%, respectively; both *p* < 0.001). Urban cyclists were significantly more frequently crossing the roadway (30.6% vs. 0.0%; *p* < 0.001) when fatally struck. One-third of urban CMVC occurred at very low speed (15 km/h or less) compared to none in a rural environment (33% vs. 0%; *p* < 0.001). Urban cyclist fatalities were significantly more likely to be on a bike lane or shared path (28.6% vs., 0%; *p* < 0.001; with 9/14 of them, 64.3%, struck by a heavy truck while using this cycling infrastructure), struck by a heavy vehicle (30.6% vs. 6.3%; *p* = 0.006), turning (26.5% vs. 2.9%; *p* = 0.005) and runover/dragged (49.0% vs. 8.8%; *p* < 0.001).

Cyclists in fatal collisions in rural areas were older than urban cyclist fatalities with a median (IQR) age of 49.0 (33.25-62.0) years compared to 44.0 (24.0-56.5; *p* = 0.345). Rural collisions were associated with a significantly higher median estimated impact speed of 80 (60–80) km/h (vs. 50 [15-72.5]km/h), as well as a higher proportion of crashes at high speeds in excess of 50 km/h (94.1% vs. 49.0%; *p* < 0.001) (Table 1). Cyclists in rural areas were significantly more often riding on roadways with traffic (55.9% vs. 16.3%; *p* < 0.001) or on the shoulder of the road (23.5% vs. 6.1%); *p* = 0.044) in dark conditions (44.1% vs. 4.1%; *p* < 0.001). No rural CMVC had a sidewalk or bike lane/share path available (0.0% vs. 83.7%; 0.0% vs. 32.7%; *p* < 0.001).

## Discussion

This descriptive study examined the epidemiology of cycling fatalities in Ontario over a 7-year period and found 83 unintentional, predominantly male cyclist fatalities, most often occurring in the summer months, during daylight hours. The majority of cyclists were not wearing a helmet at the time of their fatal collision resulting in the head as the most severely injured body region. Possible distraction from the use of headphones and cell phones, along with alcohol and drug impairment, were other contributing factors in these cyclist fatalities. Environmental characteristics including urban and rural environments, cycling infrastructure and other aspects of the built environment can increase the risk of collisions and sustaining injuries [15]. Our analysis identified different risk factors and associations based on crash location including speed, intersections, turning with the involvement of heavy vehicles, lighting conditions, and cycling infrastructure, which will be discussed herein.

Our epidemiologic description of fatal cycling collisions is consistent with the previously reported literature from North America and other developed countries [2, 24–27]. Males have been reported to have a higher likelihood of sustaining more severe injures compared to female cyclists [27]. Our fatalities had a 4:1 male: female ratio, consistent with the 81% to 88% male cyclist fatalities reported in earlier studies [2, 24–26], with the adults aged 35 to 64 years being the most common age group of cyclists [2]. Previous research reported a median age of 47 years [25], similar to the median age of 48 years found in our study. Confirming these demographics of cyclist fatalities in our geographic regions is vital in defining the population that prevention initiatives need to be targeted to, but to determine what those countermeasures should be, a safe system approach to cycling safety is needed to help reduce serious cycling injury and deaths, aligning with Vision Zero action plans throughout the world.

Vision Zero is a strategy to eliminate traffic fatalities and severe injuries. Initiated in Sweden in 1997, it has since become a global, long-term strategic road safety goal for all road users [17, 18, 28–30]. It offers a paradigm for the reduction cyclist deaths through a safe system approach, while promoting safe, equitable mobility options for all [29]. A safe system approach recognizes that people make mistakes, and focuses on a shared responsibility for influencing system-wide practices, policies, and designs to roadways and vehicles to lessen the occurrence and severity of crashes [17]. In order to determine the recommendations to change practice for the prevention of cyclist serious injury and deaths, regional risk factors associated with these CMVC need to be identified.

Previous research has established bike lanes, the use of alcohol, available lighting or riding at night, vehicle speed, location of crash, and the use of helmets as factors found to influence the level of injury severity of CMVC [31–33]. These factors were also identified in our analysis associated with cycling fatalities which need to be addressed through layered countermeasures for safer roads, safer speed and safer vehicles as part of a safe system approach to mitigate risk and reduce serious cycling injuries and fatalities [18]. 

Almost two-thirds of cyclists in our study were not wearing a helmet at the time of their fatal collision resulting in nearly all cyclists sustaining a severe head injury. The efficacy of bicycle helmets has long been established, with the use of bicycle helmets estimated to reduce the risk of head injury for cyclists by 60% and brain injury by 58% [34]. A more recent meta-analysis confirmed these results, finding helmet use resulted in an estimated head injury reduction of 48% among all age groups [35]. The lack of helmet use may have contributed to sustaining a fatal injury for the unhelmeted cyclists in our study, as it was the most severely injured body region, similar to a recent national study of cyclists [4]. Worldwide, 28 countries have been identified as having bicycle helmet legislation, with most countries applying this legislation to children and adolescents below a certain age [36]. In the US, there are 22 states, the District of Columbia and more than 200 local jurisdictions that have bicycle helmet laws in effect [37]. In Canada, approximately two-thirds (8 out of 13) of provinces and territories have bicycle helmet legislations: 5 for all ages, and 3 for less than 18 years of age [38]. There are opportunities for improvement with helmet legislation, enforcement, and incentives to promote helmet use among cyclists, a known effective intervention to reduce head injuries and save lives, particularly for CMVC.

Distractions and unsafe, risky cycling behaviours are both common risk factors contributing to fatalities and severe injuries, regardless of environment [27, 39, 40]. Given the extreme physical vulnerability of cyclists on the roadways with traffic, distraction substantially increases the odds of suffering a severe injury or death [40]. During police and crash investigation of our cyclist deaths, headphones or cell phones were found at the scene or on the deceased’s body, as reported in the post-mortem, in nearly 10% of CMVC that may have contributed to the crash. Cycling can be distracted by technology, especially the use of cellphones, headphones, and navigators [40]. A study in New York City found rates of technology-related distraction were relatively low, with headphone/earbud use being the most prevalent type of distraction device observed in 2–6% of cyclists. Talking on your cell phone or looking at the screen was less common noted in < 1% of cyclists [41]. Other distractions, while more difficult to quantify, can occur for cyclists, particularly in busy, urban areas [40]. Avenues for countermeasures include bicycle safety policies, legislation and health promotion/social media campaigns to raise public awareness on the dangers of distractions and using technology while cycling [41]. 

Another cyclist high risk behaviour found in our study in both urban and rural areas, was the use of drugs and alcohol. These substances impair the cyclists’ physical ability to operate their bicycle, along with the negative effects on human perception and decision-making, increasing the likelihood of crashing and sustaining severe or even fatal injuries [27]. Previous research has demonstrated positive associations with alcohol and/or drug use for severe and fatal collisions involving cyclists and other VRU [2, 24, 25, 31, 42]. Nearly one-quarter of our cyclist deaths had a positive toxicology report with THC as the most common drug, followed by multiple drugs and/or alcohol. This is consistent with previous reports demonstrating drugs at a higher level of involvement than alcohol among VRU, but to a higher level than in our study, with 43.4% and 39.7% of VRU testing positive for drugs and alcohol, respectively [12]. A study of fatal and serious VRU injuries in Canada found higher rates of positive drug use for bicyclists (31.4%), than for alcohol with 25.2% and 9.0% of fatally injured male and female cyclists having consumed alcohol, respectively. Of drinking cyclist fatalities, over three-quarter (76.5%) had BAC over 80 mg/dL [42]. As with other risky and unsafe behaviours of cyclists, implementation of prevention initiatives such as public health/social media campaigns relating to the dangers of impairment is recommended, as well as enforcement of current legislation and the development of stricter legislation and policies [25]. 

### Urban versus rural cyclist fatalities

Our analysis also found significant differences in risk factors associated with cyclist fatalities depending on whether the collision occurred in either an urban or a rural environment. Urban areas are characterized as communities with higher population density, more land use mix, and greater connectivity, which has been found to be correlated to active transportation such as walking and cycling [43]. A Swedish study found more than 80% of serious injuries on urban road involved cyclist and pedestrians [28]. Factors including socioeconomic status, ethnicity, and the built environment (including cycling infrastructure and street lighting, for example) differ in urban and rural areas which can have an impact on the likelihood of crashing and sustaining injuries [15, 26, 44]. As a result, examining the urban and rural differences in cycling road traffic fatalities is necessary to better understand, target and implement prevention strategies within these very different types of environments [44]. 

#### Urban Environment 

Urban cyclist fatalities had significantly more collisions in an intersection, on a bike lane/share path, and while crossing the roadway. Cyclists in the urban environment had significantly more vehicles slowed or stopped, often making a turn, resulting in being struck more often at a very low speed. There was also a higher likelihood of involving a heavy truck and being swept under the vehicle and run over. Our findings are expected given dense urban environments with people biking or walking as their primary form of transportation, sharing the roadway with vehicles in congested areas on streets with high levels of traffic. Urban arterial roads are designed with more lanes, intersections, crosswalks, bike lanes, traffic signals, and are closer to business areas, resulting in higher safety risks of crossing behaviours [27]. 

One of the most critical and common crash configurations in urban areas occurs when motorists turn right at an intersection and cyclists cross the road, often resulting in severe injuries for cyclists [45, 46]. The consequences are more dire when a heavy vehicle is involved due to the design of large trucks that presents inherent safety challenges including blind spots on trucks and the common occurrence of ‘side underride’, when a pedestrian or cyclist is swept under the rear tires of a truck after side impact and runover [47]. We found this occurred in our urban cyclist fatalities, with nearly one-third of urban CMVC fatalities involving heavy trucks. Large vehicles, including buses, waste disposal and utility trucks, make up a small proportion of vehicles on urban streets, but are disproportionately involved in fatal crashes, particularly involving cyclists and pedestrians [47]. For example, in NYC, large trucks comprise 3.6% of the traffic vehicles, yet they are involved in 32% of cyclist fatalities, similar to our results [47]. Utilizing a safe system lens, several layers of safety interventions can be implemented to collectively address this safety issue for cyclists and other VRU. Suggested interventions include driver training, education, and policy changes at the municipality level including restricting access to large vehicles on streets that prioritize active modes of transportation like walking and cycling, such as near schools [47, 48]. Additionally, design features and safety devices on the vehicle that can be retroactively installed on large trucks such as cross-over and convex mirrors, cameras and side guards, which have demonstrated success in averting underride incidences and decreasing fatalities [47]. 

New technology can also be designed into vehicles including Advanced Driver Assistance Systems (ADAS). A recent study found that drivers considerably reduced their speed and stopped their vehicle further away from the intersection when a VRU was present [49]. ADAS that warns drivers about the presence of a cyclist traveling in parallel direction (i.e., Blind Spot Information Systems, BLIS) or turn collision warning system for heavy commercial vehicles can detect VRU in the vehicle’s blind spots and alert the driver before making a turn, allowing adequate time for the driver to react and take action to prevent collisions [50]. Another ADAS feature that can intervene to avoid a collision with a cyclist or a pedestrian is the Automatic Emergency Braking Systems (AEBS) triggered to stop the vehicle when a VRU is detected. These design features may play a crucial role in reducing cyclist fatalities in urban environments, where the risk of CMVC is heightened.

Improving roadway infrastructure design such as traffic calming measures including pedestrian islands and curb extensions have been found to be effective, with a focus on ensuring infrastructure changes are targeted to high-risk intersections in urban areas [26]. A recent study in NYC found prioritizing traffic calming at intersections that abut long street segments to be the most effective to prevent high speed crashes and fatalities that are more likely on longer roads [51]. Additionally, improved traffic light sequence and installing crosswalks for cyclists away from intersections can help separate and control the movement of cyclists and vehicles in space and time on the roadways. Utilizing urban planning and roadway design has the potential to reduce the occurrence of the dangerous intersection interactions between cyclists and all vehicles, large and small [47, 48, 51]. 

While cycling infrastructure including bike lanes, or cycle tracks, for dedicated lanes with a physical separation or barrier between cyclists and motor vehicles have been found to increase safety for cyclist [5], in our study several cyclists were killed in the bike lane. However, all 8 of these cyclists were struck by a heavy vehicle while cycling in a bike lane. The hazards associated with heavy vehicles, particularly when turning right, often through the bike lane, outweigh the protective effect of the cyclist tracks. Cyclists need to be aware of these dangers and not get a false sense of security while using them.

While not present in our study population, an emerging trend that needs to be considered when designing roadways and active transportation infrastructure is the growing use of micromobility devices, particularly in the urban environment. These electrical vehicles (EV) including e-scooter, e-bikes and hoverboards have gained popularity as a sustainable, relatively low-cost urban transportation personal device and shared device through vehicle sharing platforms now available in many major North American cities and pose a new risk to pedal cyclists [52, 53]. Ridership has increased more than 50-fold in the past decade and with that a surge in ED visits for injuries, increases as high as 600% in recent years reported, due in part to the fast acceleration of these machines with novice drivers [52, 53]. E-bikes and e-scooters have been found to have differences in characteristics of riders, risk factors, use of the vehicle and injury patterns than traditional bicycles [52, 54, 55]. EV riders were more likely to engage in risky behaviours like intoxication and riding without a helmet, with e-bike-related injuries to be more than three times more likely than pedal bicycles to involve a collision with a pedestrian (OR = 3.3, 95% CI 0.5 to 23.6) [56]. EV sharing bike lanes or roadways with traditional bicycles and pedestrians pose a threat to the safety of these other VRU. It has been suggested that changes to active transportation infrastructure (i.e., traffic calming, protected bike lanes, EV lanes, wider sidewalks, docking stations) to accommodate EV may be warranted, along with changes to education, policies and legislation for micromobility devices to ensure the safety of all road users [52, 55, 56]. 

#### Rural Environment

Cyclists face different risk factors when cycling in a rural environment. Very high speeds and dark lighting conditions, while riding on the roadway with traffic were factors significantly associated with rural cyclist fatalities. The majority of both vehicles and cyclists were going forward together on the roadway at the time of the collision, as these rural collision locations lacked cycling infrastructure, which is often the case in rural areas [27]. Previous research has demonstrated decreases in CMVC rates after the installation of cycle tracks in streets previously without cycling infrastructure, up to 70% in one Canadian study [5, 57]. This effectiveness of bike lanes/cycle tracks may be attributed to physical separation, allowing for an increased distance between cyclists and motor vehicles [5]. Given their protective benefit, rural areas could benefit from this type of cycle infrastructure to provide more sufficient riding space and/or a physical barrier to allow cyclists to more comfortably and safely cycle while alleviating interactions between bicycles and motor vehicles [5, 26, 27]. 

High speed is a known risk factor for CMVC [26, 33], especially on rural roads where motor vehicles and cyclists are often traveling at higher speeds than in urban areas [27]. A recent study of e-bikes quantified the fatality risk by crash impact speed. This analysis found risk of death for riders exponentially increasing from approximately 2.9% at vehicle impact speed of 30 km/h to 23% at 50 km/h, 50% at 60 km/h, and 90% at 80 km/h [58]. Given the median crash impact speed for rural cyclists in our study was 80 km/h, countermeasures for speed reductions including roundabouts and speed humps could be an effective means to mitigate the impact of high speeds on rural road [26]. Previous research has found an inequity of speed hump installation with communities with higher socioeconomic status, more engaged in the political process for advocating for their installation, having more of these traffic calming measures installed on their roadways [59]. Selection of sites for traffic calming measures should be based on need, in high crash-risk areas, in both rural and urban communities.

**Fig. 3:**
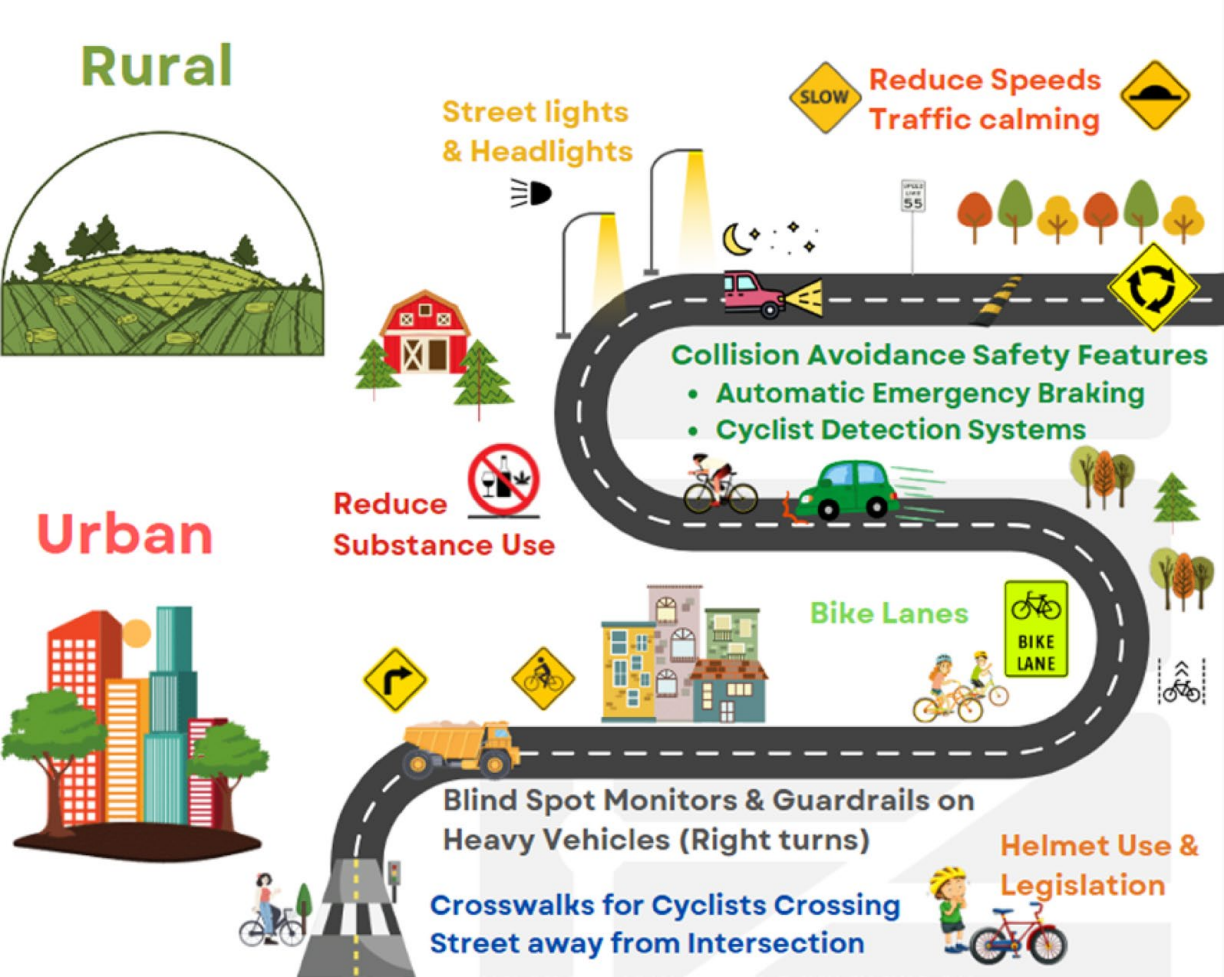
Schematic of countermeasures in the rural and urban environments for the reduction of cycling collisions, serious injuries and deaths

Given that nearly half of rural crashes occurred in dark conditions, lack of visibility of the cyclist was a significant factor contributing to CMVC in our study. Over three-quarters of rural cyclists in our analysis had a lack of conspicuity. Public awareness, policy, or legislative changes surrounding the use of reflective clothing or bike lights would increase cyclists’ visibility and may save lives. Because a safe system approach embodies a shared responsibility [17, 18, 60], the influences of other factors such as roadway and vehicle should be considered in addition to the cyclists’ behaviour and responsibility to be visible. This includes the installation of rural street lighting, particularly at curves in the roads or areas identified as high-risk [27]. Higher performance vehicle headlights are another vehicle design strategy as vehicle headlights are the primary method of increasing the illumination available to drivers at night to improve visibility and allow them to see VRU and other objects on the side of the roadway, such as deer and other wildlife, common alongside rural roadways [61, 62]. These lighting strategies for roadways and vehicles are recommended to improve driver and cyclist visibility, especially in rural environments that more often have CMVC in dark conditions. A schematic of the countermeasures suggested in the rural and urban environments for the reduction of cycling collisions, serious injuries and deaths is presented in Fig. 3.

A limitation of this study is the small sample size of 83 cyclist fatalities, which raises concern of the representativeness of the sample. However, our study population consists of all cyclist fatalities that had a postmortem performed at the Office of the Chief Coroner in Ontario, comprising over half (59%) of all cyclist fatalities in the province. While there is a relatively small number of deaths in this study, they represent the majority of cycling fatalities in the region. This makes it highly likely that our results resemble what would be expected in the target population and therefore, increase their generalizability [63]. To provide further support for this notion, our study population was found to be representative of other CMVC fatality studies in the literature with similar reported characteristics and risk factors [2, 25, 26, 64]. Despite this, our study may not represent cyclist deaths in all other regions, or less severe CMVC as only fatalities were included in these analyses. So, prior to implementing a prevention initiative for the reduction of CMVC, injuries and deaths, it is recommended that investigators utilize their own local data from urban and rural areas in their region to determine if the same risk factors are present in their communities to determine the appropriateness of interventions presented herein.

## Conclusion

Cycling in traffic puts cyclists at risk for severe injury and death, in both urban and rural environments. A safe system approach recognizes that people are vulnerable and inevitably make mistakes, while promoting a shared safety responsibility. Urban planners, public health advocates and road safety specialists need to develop multiple layers of passive and active safety systems to protect cyclists from death in CMVC [26]. Incorporating engineering countermeasures into the design of roadways to separate the cyclist from the vehicle, lighting in rural areas, and traffic calming measures help to reduce cyclists’ risk. Vehicle safety features include guard rails and cameras on heavy vehicles, higher rated vehicle headlight performance, along with ADAS to detect cyclists in blind spots or AEBS may be able to play important roles in the prevention of CMVC. Public health campaigns to raise awareness, as well as policy and legislative action for substance use and technology-related distractions can potentially improve the safety of the transportation system by mitigating crash risk. To be equitable, it is suggested that these countermeasures, essential for improving cyclists’ safety, be implemented in all high-risk areas, including urban and rural environments.

## Supplementary Information

Supplementary Material 1

## Data Availability

All data used and analyzed during this study are included in this published article in Additional file 1.

## Abbreviations

ADAS: Advanced Driver Assistance Systems
AEBS: Automatic Emergency Braking Systems
AIS: Abbreviated Injury Scale
BAC: Blood Alcohol Concentration
BLIS: Blind Spot Information Systems
CMVC: Cyclist and Motor Vehicle Collision
IBM®: International Business Machines Corporation
IQR: Interquartile Range
ISS: Injury Severity Score
Km/h: Kilometers per hour
LTV: Light Trucks and Vans
OCC: Office of the Chief Coroner
SPSS®: Statistical Package for Social Sciences
SUV: Sports Utility Vehicle
THC: Tetrahydrocannabinol
VRU: Vulnerable Road User

## Declarations

### About this supplement

This article has been published as part of Injury Epidemiology Volume 12 Supplement 1, 2025: Proceedings of the 29th Annual Injury Free Coalition for Kids^®^ Conference: Forging New Frontiers: Engineering for Equity. The full contents of the supplement are available online at https://injepijournal.biomedcentral.com/articles/supplements/volume-12-supplement-1.

## References

[1] World Health Organization. Cyclist safety: An information resource for decision-makers and practitioners [Internet]. Geneva. 2020 [cited 2025 Feb 12]. Available from: https://iris.who.int/bitstream/handle/10665/336393/9789240013698-eng.pdf

[2] O’Hern S, Oxley J. Fatal cyclist crashes in Australia. Traffic Inj Prev. 2018;19(sup2). 10.1080/15389588.2018.1497166 30335520

[3] Failing GRL, Klamer BG, Gorham TJ, Groner JI. The impact of the COVID-19 pandemic on pediatric bicycle injury. Int J Environ Res Public Health. 2023. 10.3390/ijerph20085515. 10.3390/ijerph20085515 PMC10139432 37107797

[4] Johnson CA, Newton WN, LaRochelle L, Daly CA. National incidence and trends of bicycle injury. J Orthop Res. 2023;41(7). 10.1002/jor.25489 36541024

[5] Ling R, Rothman L, Cloutier MS, Macarthur C, Howard A. Cyclist-motor vehicle collisions before and after implementation of cycle tracks in Toronto, Canada. Accid Anal Prev. 2020. 10.1016/j.aap.2019.105360. 10.1016/j.aap.2019.105360 31785479

[6] National Highway Traffic Safety Administration, Department of Transportation. Early Estimate of Motor Vehicle Traffic Fatalities in 2022 (Crash-Stats Brief Statistical Summary. Report No. DOT HS 813 428) [Internet]. Washington, DC. 2023 Apr [cited 2023 Sep 20]. Available from: http://crashstats.nhtsa.dot.gov/Api/Public/ViewPublication/813428

[7] Rothman L, Schwartz N, Cloutier MS, Winters M, Macarthur C, Hagel BE, et al. Child pedestrian and cyclist injuries, and the built and social environment across Canadian cities: the child active transportation safety and the environment study (CHASE). Inj Prev. 2022. 10.1136/injuryprev-2021-044459. 10.1136/injuryprev-2021-044459 PMC9340017 35058306

[8] Transport Canada. Canadian Motor Vehicle Traffic Collision Statistics: 2022 [Internet]. 2024 [cited 2025 Feb 12]. Available from: https://tc.canada.ca/en/road-transportation/statistics-data/canadian-motor-vehicle-traffic-collision-statistics-2022

[9] Beck LF, Dellinger AM, O’Neil ME. Motor vehicle crash injury rates by mode of travel, united states: using exposure-based methods to quantify differences. Am J Epidemiol. 2007;166(2). 10.1093/aje/kwm064 17449891

[10] Nilsson P, Stigson H, Ohlin M, Strandroth J. Modelling the effect on injuries and fatalities when changing mode of transport from car to bicycle. Accid Anal Prev. 2017. 10.1016/j.aap.2016.12.020. 10.1016/j.aap.2016.12.020 28086080

[11] Pucher J, Dijkstra L. Promoting safe walking and cycling to improve public health: lessons from the Netherlands and Germany. Am J Public Health. 2003. 10.2105/AJPH.93.9.1509. 10.2105/ajph.93.9.1509 PMC1448001 12948971

[12] National Center for Statistics and Analysis. Bicyclists and other cyclists: 2022 data (Traffic Safety Facts) [Internet]. Washington, D.C. 2024 Jul [cited 2025 Feb 13]. Available from: https://rosap.ntl.bts.gov/view/dot/78005

[13] Hallum SH, Chupak AL, Thomas KM, Looney EN, Witherspoon E, Huynh NH et al. Disparities in pedestrian and cyclist crashes by social vulnerability across South Carolina. J Phys Act Health. 2025;1–11. 10.1123/jpah.2024-0552 39919723

[14] Diez Roux AV, Mair C. Neighborhoods and health. Ann N Y Acad Sci. 2010. 10.1111/j.1749-6632.2009.05333.x. 10.1111/j.1749-6632.2009.05333.x 20201871

[15] Yu CY. Environmental supports for walking/biking and traffic safety: income and ethnicity disparities. Prev Med (Baltim). 2014. 10.1016/j.ypmed.2014.06.028. 10.1016/j.ypmed.2014.06.028 24979334

[16] Gilna GP, Stoler J, Saberi RA, Baez AC, Ramsey WA, Huerta CT, et al. Analyzing pediatric bicycle injuries using geo-demographic data. J Pediatr Surg. 2022. 10.1016/j.jpedsurg.2021.12.034. 10.1016/j.jpedsurg.2021.12.034 35109994

[17] Vison Zero Network. Demystifying the Safe System Approach | Vision Zero Network [Internet]. 2023 [cited 2025 Feb 17]. Available from: https://visionzeronetwork.org/resources/demystifying-the-safe-system-approach/

[18] Arason N. The Safe Systems Approach for Road Safety | Vision Zero Canada [Internet]. Transportation Talk - Winter 2018-19. 2019 [cited 2025 Feb 17]. pp. 11–6. Available from: https://visionzero.ca/the-safe-systems-approach-for-road-safety/

[19] Wegman F, Schepers P. Safe system approach for cyclists in the netherlands: towards zero fatalities and serious injuries? Accid Anal Prev. 2024;195. 10.1016/j.aap.2023.107396 38043211

[20] Transport Canada. Crash database. Ottawa: Transport Canada; 2019.

[21] Association for the Advancement of Automotive Medicine. Abbreviated injury scale 2015. Barrington: American Association for the Advancement of Automotive Medicine; 2015.

[22] Google. Google Maps [Internet]. 2023 [cited 2023 Sep 24]. Available from: https://www.google.ca/maps

[23] Statistics Canada. Urban and rural areas - Urban versus rural variant [Internet]. Statistics Canada Statistical Classifications. 2019 [cited 2025 Feb 18]. Available from: https://www23.statcan.gc.ca/imdb/p3VD.pl?Function=getVD%26TVD=113331%26CVD=113332%26CLV=0%26MLV=2%26D=1%26adm=0%26dis=0

[24] National Center for Statistics and Analysis. Bicyclists and other cyclists: 2019 data (Traffic Safety Facts) [Internet], Washington DC. 2021 Oct [cited 2025 Feb 10]. Available from: https://crashstats.nhtsa.dot.gov/Api/Public/Publication/813197

[25] Gaudet L, Romanow NTR, Nettel-Aguirre A, Voaklander D, Hagel BE, Rowe BH. The epidemiology of fatal cyclist crashes over a 14-year period in Alberta, Canada. BMC Public Health. 2015;15(1). 10.1186/s12889-015-2476-9 PMC4650295 26577650

[26] Mason-Jones AJ, Turrell S, Gomez GZ, Tait C, Lovelace R. Severe and fatal cycling crash injury in Britain: time to make urban cycling safer. J Urban Health. 2022. 10.1007/s11524-022-00617-7. 10.1007/s11524-022-00617-7 PMC8916078 35277814

[27] Du B, Zhang C, Sarkar A, Shen J, Telikani A, Hu H. Identifying factors related to pedestrian and cyclist crashes in ACT, Australia with an extended crash dataset. Accid Anal Prev. 2024;207:107742. 39137657 10.1016/j.aap.2024.107742

[28] Värnild A, Tillgren P, Larm P. What types of injuries did seriously injured pedestrians and cyclists receive in a Swedish urban region in the time period 2003–2017 when vision zero was implemented? Public Health. 2020. 10.1016/j.puhe.2019.11.019. 10.1016/j.puhe.2019.11.019 31954870

[29] Webber BJ, Whitfield GP, Rose KM, Stowe EW, Zaganjor H, Ederer DJ, et al. Prevalence of vision zero action plans or strategies: USA, 2021. Inj Prev. 2024. 10.1136/ip-2023-044926. 10.1136/ip-2023-044926 PMC11137438 38378255

[30] He Y, Fan Y, Yan L, Peng J, Li Z. Visualization and analysis of global vision zero studies and policy orientation in China. 19, Int J Environ Res Public Health. 2022. 10.3390/ijerph192214841 PMC9690604 36429560

[31] Helak K, Jehle D, McNabb D, Battisti A, Sanford S, Lark MC. Factors influencing injury severity of bicyclists involved in crashes with motor vehicles: bike lanes, alcohol, lighting, speed, and helmet use. South Med J. 2017. 10.14423/SMJ.0000000000000665. 10.14423/SMJ.0000000000000665 28679010

[32] Boufous S, De Rome L, Senserrick T, Ivers R. Risk factors for severe injury in cyclists involved in traffic crashes in Victoria, Australia. Accid Anal Prev. 2012. 10.1016/j.aap.2012.03.011. 10.1016/j.aap.2012.03.011 23036419

[33] Embree TE, Romanow NTR, Djerboua MS, Morgunov NJ, Bourdeaux JJ, Hagel BE. Risk factors for bicycling injuries in children and adolescents: a systematic review. Pediatrics. 2016. 10.1542/peds.2016-0282. 10.1542/peds.2016-0282 27940760

[34] Attewell RG, Glase K, McFadden M. Bicycle helmet efficacy: a meta-analysis. Accid Anal Prev. 2001;33(3):345. 11235796 10.1016/s0001-4575(00)00048-8

[35] Høye A. Bicycle helmets – to wear or not to wear? A meta-analyses of the effects of bicycle helmets on injuries. Accid Anal Prev. 2018. 10.1016/j.aap.2018.03.026. 10.1016/j.aap.2018.03.026 29677686

[36] Esmaeilikia M, Grzebieta R, Olivier J. A systematic review of bicycle helmet laws enacted worldwide. J Australas Coll Road Saf. 2018;29(3).

[37] National Safety Council. Bicycle Deaths [Internet]. Injury Facts. 2025 [cited 2025 Feb 12]. Available from: https://injuryfacts.nsc.org/home-and-community/safety-topics/bicycle-deaths/

[38] Parachute. Bicycle Helmet Canadian Legislation Chart [Internet]. 2019 [cited 2025 Feb 12]. pp. 1–2. Available from: https://parachute.ca/wp-content/uploads/2019/08/Bicycle-Helmet-Canadian-Legislation-Chart.pdf

[39] Wang C, Zhang W, Feng Z, Wang K, Gao Y. Exploring factors influencing the risky cycling behaviors of young cyclists aged 15–24 years: a questionnaire-based study in China. Risk Anal. 2020. 10.1111/risa.13499. 10.1111/risa.13499 32367568

[40] Useche SA, Alonso F, Montoro L, Esteban C. Distraction of cyclists: how does it influence their risky behaviors and traffic crashes? PeerJ. 2018;2018(9). 10.7717/peerj.5616 PMC6139010 30225181

[41] Ethan D, Basch CH, Johnson GD, Hammond R, Chow CM, Varsos V. An analysis of technology-related distracted biking behaviors and helmet use among cyclists in New York City. J Community Health. 2016. 10.1007/s10900-015-0079-0. 10.1007/s10900-015-0079-0 26323983

[42] Vanlaar W, Mainegra Hing M, Brown S, McAteer H, Crain J, McFaull S. Fatal and serious injuries related to vulnerable road users in Canada. J Saf Res. 2016. 10.1016/j.jsr.2016.07.001. 10.1016/j.jsr.2016.07.001 27620936

[43] Saelens BE, Sallis JF, Frank LD. Environmental correlates of walking and cycling: findings from the transportation, urban design, and planning literatures. Ann Behav Med. 2003. 10.1207/S15324796ABM2502_03. 10.1207/S15324796ABM2502_03 12704009

[44] Carlson SA, Whitfield GP, Peterson EL, Ussery EN, Watson KB, Berrigan D, et al. Geographic and urban–rural differences in walking for leisure and transportation. Am J Prev Med. 2018. 10.1016/j.amepre.2018.07.008. 10.1016/j.amepre.2018.07.008 PMC9619131 30344032

[45] Saul H, Junghans M, Dotzauer M, Gimm K. Online risk Estimation of critical and non-critical interactions between right-turning motorists and crossing cyclists by a decision tree. Accid Anal Prev. 2021;163. 10.1016/j.aap.2021.106449 34749268

[46] Buch TS, Jensen SU. Incidents between straight-ahead cyclists and right-turning motor vehicles at signalised junctions. Accid Anal Prev. 2017. 10.1016/j.aap.2016.07.035. 10.1016/j.aap.2016.07.035 27491717

[47] Vision Zero Network. How can cities increase the safety of large vehicles in urban areas? [Internet]. 2023 [cited 2025 Feb 27]. Available from: https://visionzeronetwork.org/resource/how-can-cities-increase-the-safety-of-large-vehicles-in-urban-areas/

[48] Kircher K, Ahlström C. Truck drivers’ interaction with cyclists in right-turn situations. Accid Anal Prev. 2020;142. 10.1016/j.aap.2020.105515 32380238

[49] Schindler R, Bianchi Piccinini G. Truck drivers’ behavior in encounters with vulnerable road users at intersections: results from a test-track experiment. Accid Anal Prev. 2021;159. 10.1016/j.aap.2021.106289 34340136

[50] BOSCH. Turn Collision Warning for Heavy Commercial Vehicles [Internet]. 2025 [cited 2025 Mar 1]. Available from: https://www.bosch-mobility.com/en/solutions/assistance-systems/turn-collision-warning-cv/

[51] Roberts LE, Bushover B, Mehranbod CA, Gobaud AN, Fish C, Eschliman EL, et al. Physical environmental roadway interventions and injury and death for vulnerable road users: a natural experiment in new York City. Inj Prev. 2024;24:ip–2023. 10.1136/ip-2023-045219 PMC11771210 38789249

[52] Fernandez AN, Li KD, Patel HV, Allen IE, Ghaffar U, Hakam N, et al. Injuries with electric vs conventional scooters and bicycles. JAMA Netw Open. 2024;7(7):e2424131. 39042404 10.1001/jamanetworkopen.2024.24131 PMC11267411

[53] Lopez D, Lin T, Vollaro A, Arcoleo KJ, Mello MJ. Trends and Costs of Non-Fatal Micromobility-Related Injuries Treated in Emergency Departments in Rhode Island, 2016–2021. R I Med J (2013). 2024;107(10):18–22. PMC11570986 39331008

[54] Benhamed A, Gossiome A, Ndiaye A, Tazarourte K. Characteristics and comparison between e-scooters and bicycle-related trauma: a multicentre cross-sectional analysis of data from a road collision registry. BMC Emerg Med. 2022;22(1):164. 36175859 10.1186/s12873-022-00719-0 PMC9520117

[55] Burford KG, Itzkowitz NG, Rundle AG, DiMaggio C, Mooney SJ. The burden of injuries associated with E-bikes, powered scooters, hoverboards, and bicycles in the United States: 2019–2022. Am J Public Health. 2024;114(12):1365–74. 39265126 10.2105/AJPH.2024.307820 PMC11540957

[56] DiMaggio CJ, Bukur M, Wall SP, Frangos SG, Wen AY. Injuries associated with electric-powered bikes and scooters: analysis of US consumer product data. Inj Prev. 2019. 10.1136/injuryprev-2019-043418. 10.1136/injuryprev-2019-043418 31712276

[57] Lusk AC, Furth PG, Morency P, Miranda-Moreno LF, Willett WC, Dennerlein JT. Risk of injury for bicycling on cycle tracks versus in the street. Inj Prev. 2011. 10.1136/ip.2010.028696. 10.1136/ip.2010.028696 PMC3064866 21307080

[58] Hu L, Hu X, Wang J, Kuang A, Hao W, Lin M. Casualty risk of e-bike rider struck by passenger vehicle using China in-depth accident data. Traffic Inj Prev. 2020. 10.1080/15389588.2020.1747614. 10.1080/15389588.2020.1747614 32297809

[59] Rothman L, Cloutier MS, Manaugh K, Howard AW, MacPherson AK, MacArthur C. Spatial distribution of roadway environment features related to child pedestrian safety by census tract income in Toronto, Canada. Inj Prev. 2020. 10.1136/injuryprev-2018-043125. 10.1136/injuryprev-2018-043125 30936120

[60] National Transportation Safety Board. Protect Vulnerable Road Users Through a Safe System Approach [Internet]. 2021–2023 NTSB Most Wanted List of Transportation Safety Improvements. 2022 [cited 2023 Sep 20]. Available from: https://www.ntsb.gov/Advocacy/mwl/Pages/mwl-21-22/mwl-hs-02.aspx

[61] Brumbelow ML. Light where it matters: IIHS headlight ratings are correlated with nighttime crash rates. J Saf Res. 2022. 10.1016/j.jsr.2022.09.013. 10.1016/j.jsr.2022.09.013 36481031

[62] Vanlaar WGG, Gunson KE, Brown SW, Robertson RD. Wildlife-Vehicle Collisions in Canada: A Review of the Literature and a Compendium of Existing Data Sources [Internet]. Ottawa; 2012 Aug [cited 2025 Mar 4]. Available from: https://tirf.ca/wp-content/uploads/2017/01/WildlifeVehicle_Collision_Deliverable1_Eng_6.pdf

[63] Rudolph JE, Zhong Y, Duggal P, Mehta SH, Lau B. Defining representativeness of study samples in medical and population health research. BMJ Med. 2023. 10.1136/bmjmed-2022-000399. 10.1136/bmjmed-2022-000399 PMC10193086 37215072

[64] National Center for Statistics and Analysis. Traffic safety facts 2015 data: motorcycles. Traffic Safety Facts; 2016.

